# An investigation of Stenotrophomonas maltophilia-positive culture caused by fiberoptic bronchoscope contamination

**DOI:** 10.1186/s12879-019-4670-3

**Published:** 2019-12-21

**Authors:** Bende Liu, Shenglan Tong

**Affiliations:** 1 Department of Emergency Medicine, Union Hospital, Tongji Medical College, Huazhong University of Science and Technology, Wuhan, 430022 China; 2 Department of Clinical Laboratory, First People’s Hospital of Jiangxia District in Wuhan, Wuhan, 430200 China

**Keywords:** Stenotrophomonas maltophilia, Fiberoptic bronchoscope, Nosocomial infection

## Abstract

**Background:**

*Stenotrophomonas maltophilia* (SMA) is present in hospital environments and has been one of the pathogens that cause nosocomial contamination and infections. To investigate the occurrence of *Stenotrophomonas maltophilia* (SMA) in bronchoscope lavage fluid (BALF) among 25 cases treated in the Division of Infection and to trace the contamination source and transmission route.

**Methods:**

25 cases of SMA positive BALF occurring from May 11 to August 10, 2018 were tested for drug sensitivity. Environmental hygiene conditions were investigated to identify the source of contamination and the route of transmission.

**Results:**

BALF associated SMA was in all cases sensitive to minocycline, levofloxacin and chloramphenicol and resistant to ceftazidime and imipenem. 92.3% of samples were sensitivity to compound sulfamethoxazole. Investigation of environmental hygiene parameters revealed SMA growing on the inner wall of the fiberoptic bronchoscope as a likely source of contamination.

**Conclusion:**

Incomplete cleaning and sterilization of the fiberoptic bronchoscope led to SMA nosocomial contamination. Strict sterilization procedures are required to prevent and control nosocomial contamination.

## Background

**Fig. 1:**
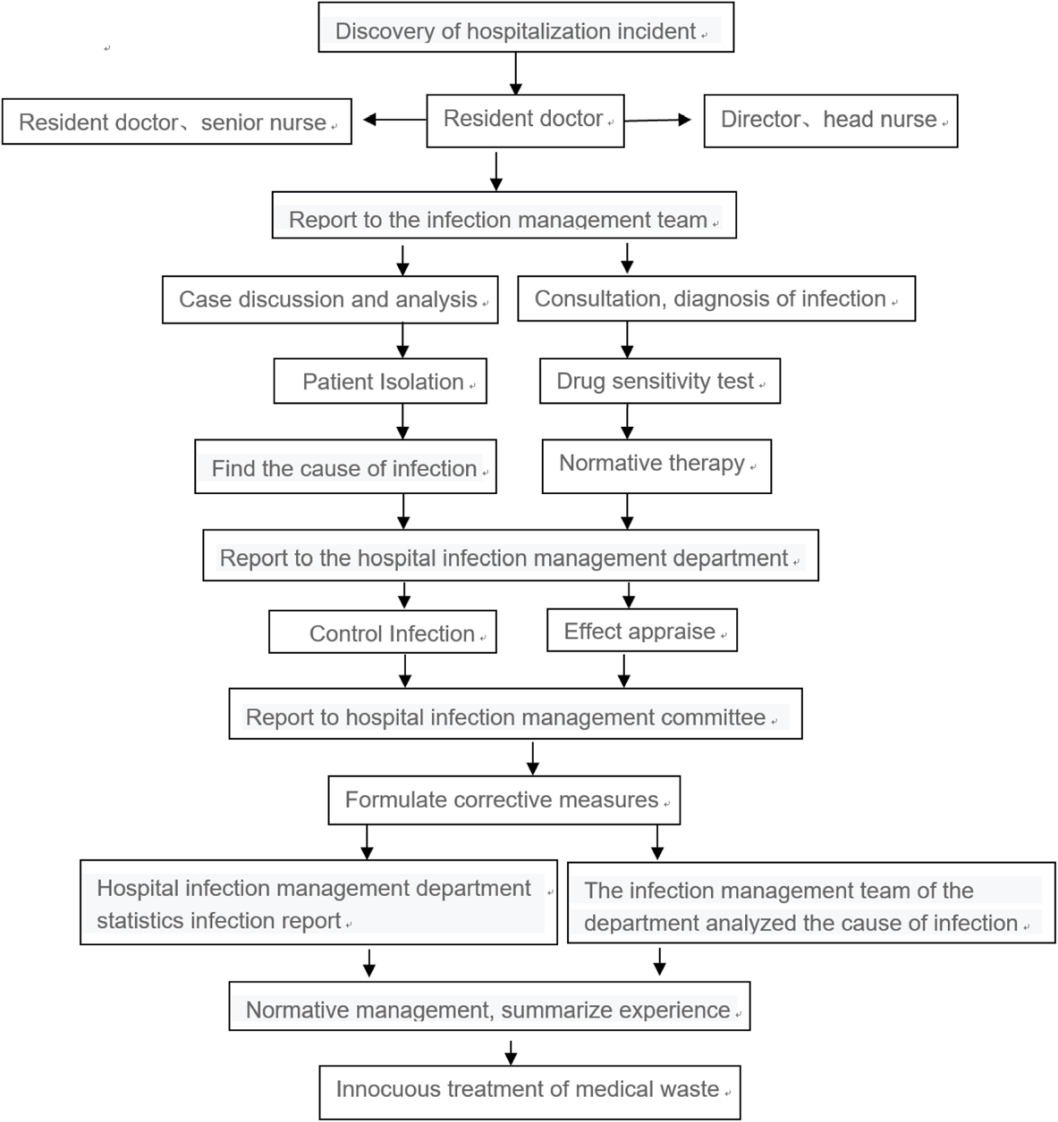
Flow chart of investigation of suspected nosocomial contamination

*Stenotrophomonas maltophilia* (SMA) is a non-fermenting gram-negative bacillus found in plants and soil and on the surface of human skin. It is present in hospital environments and is detected in the respiratory and intestinal tracts. SMA is one of the pathogens that cause nosocomial infections, superseded among non-fermenting Gram-negative bacilli only by *Pseudomonas aeruginosa* and *Acinetobacterbaumannii* [1]. SMA infection occurs frequently in hospitalized patients with low immune function, malignant tumors, hemodialysis, diabetes and those receiving immunosuppressive agents [2]. Infections lead to bacteremia, endocarditis and infections of the respiratory tract, urethra and wounds. 25 occurrences of SMA positive BALF were recorded in the Division of Infection of our institution between May 11 and August 10, 2018. None of the patients exhibited symptoms of infection. Preventive measures were instituted to protect patients and a careful investigation was undertaken to identify the source of SMA. Incomplete cleaning and sterilization of the fiberoptic bronchoscope was identified as a potential risk of nosocomial cross-contamination. Here, we report the investigative and preventive measures taken in response to the discovery of SMA among patient samples (Fig. 1) and the results of interventions.

## Methods

### Patients

Between May 11 and August 10, 2018 the Division of Infection, the First People’s Hospital of Jiangxia District in Wuhan City (tertiary general hospital), detected SMA positive BALF from 25 patients (14 male, 11 female). Patient information, disease history, the condition of invasive operations, usage of antibacterial drugs, and the results of drug sensitivity testing were compiled.

### Growth media and antibacterial drugs

Mueller-Hinton (MH) agar medium was purchased from Guangzhou Dijing Microbiology Co., Ltd.. Drug sensitivity test paper (combining sulfamethoxazole, SXT; minocycline, MH; levofloxacin, LVX; ticarcillin/clavulanic acid, TIM; ceftazidime, CAZ; chloramphenicol, CL and imipenem, IPM) was purchased from OXOID (United Kingdom).

### Bacteria isolation, identification and drug sensitivity testing

Bacteria isolation and culture were carried out according to National Clinical Laboratory Procedures. Identification was performed using the automatic bacterial identification system DL-96II at Zhuhai Dier Biological Co., Ltd.. The Kirby-Bauer paper/agar diffusion method was used to determine bacterial sensitivity to antimicrobial agents. The guidelines of the National Committee for Clinical Laboratory Standards (CLSI) were used to classify SMA strains as sensitive (S), intermediate (I) and resistant (R). Control bacterial strains used were *Escherichia coli* ATCC25922, *Pseudomonas aeruginosa* ATCC 27853 and *Staphylococcus aureus* ATCC 25923/ATCC29213.

### Bacterial sampling

A sterile cotton swab was used to collect samples from the air, fiberoptic bronchoscope, cleaning brush, lotion and hands. The samples were neutralized with an appropriate buffer. A total of 38 specimens were collected and sent to the microbiological laboratory for culture in a 35 °C incubator.

### Statistical methods

SPSS16.0 software was used for statistical analysis. The t-test was used to compare data. *p* < 0.05 was considered statistically significant.

## Results

### Case overview

**Fig. 2:**
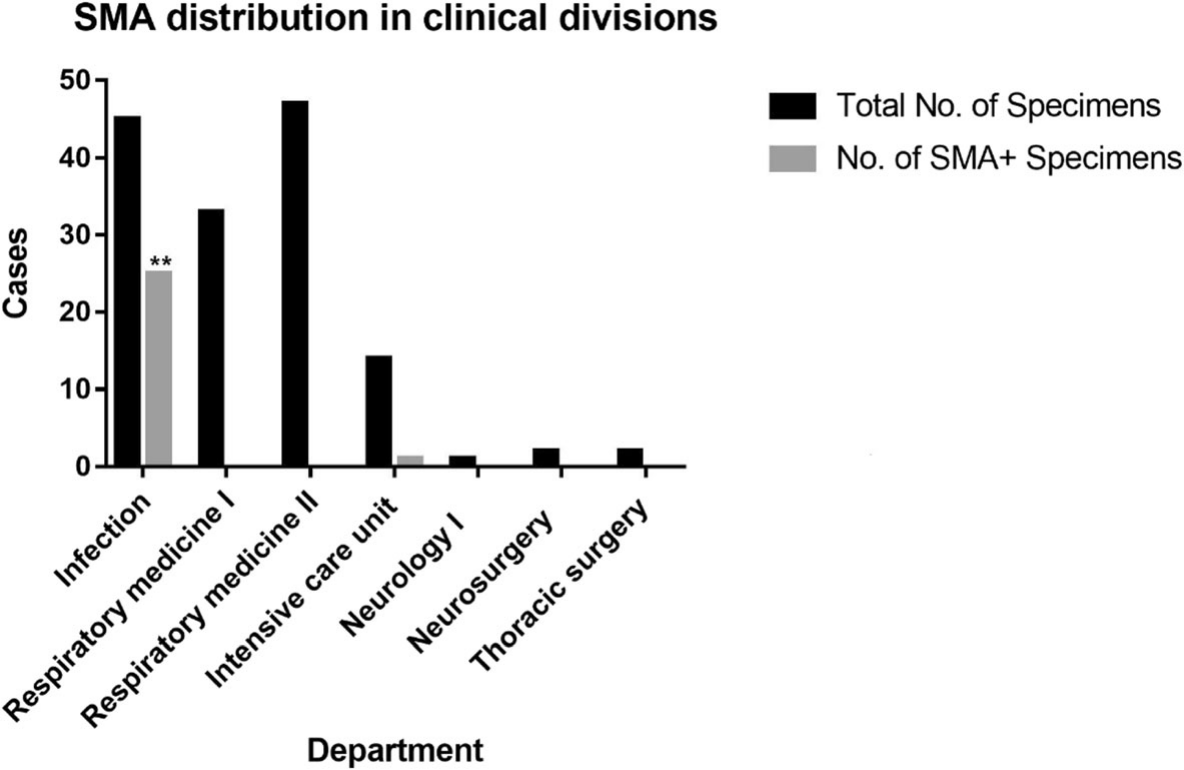
Distribution of SMA among different clinical divisions. Statistical analysis for SMA-positive numbers between the Division of Infection and other divisions. 25 cases were detected in the Division of Infection, 1 case in the ICU, but 0 in the all other divisions. The difference was statistically significant (*p* < 0.01)

In the Division of Infection between May 11 and August 10, 2018, 25 patient samples of fiberoptic bronchoscopy alveolar lavage fluid were found to contain SMA. The patients included 14 males and 11 females, ranging in age between 18 and 87 years. During the same period, the number of alveolar lavage fluid samples found to contain SMA in the Division of Respiratory Disease, ICU and other divisions were 0, 1 and 0, respectively. The incidence of cases of SMA detection in BALF was significantly higher in the Division of Infection compared to other divisions (*p* < 0.01) (Fig. 2).

### Drug sensitivity tests

As shown in Table 1, SMA was highly sensitive (100%) to minocycline, levofloxacin, and chloramphenicol; completely resistant (100%) to ceftazidime and is somewhat resistant to imipenem. Rates of resistance to compound sulfamethoxazole and ticarcillin/clavulanic acid were 7.69 and 18.18%, respectively. Sensitivity to ticarcillin/clavulanic acid was only 9.09%. This suggested that 72.73% of samples were intermediate to ticarcillin/clavulanate.

**Table 1:** SMA drug sensitivity

Drug	Strain No.	Drug resistant rate (%)	Intermediation rate (%)	Sensitivity rate (%)
SXT	25	7.69	0	92.31
MH	25	0	0	100
LVX	25	0	0	100
TIM (MIC)	11	18.18	72.73	9.09
CAZ (MIC)	11	100	0	0
CL (MIC)	11	0	0	100
IPM	25	100	0	0

Compound sulfamethoxazole (*SXT*) minocycline (*MH*) levofloxacin (*LVX*) ticarcillin/clavulanic acid (*TIM*) ceftazidime (*CAZ*) chloramphenicol (*CL*) and imipenem (*IPM*)

### Fiberoptic bronchoscope decontamination and environmental hygiene tests

Samples were collected from the disinfected fiberoptic bronchoscope, environmental sanitation equipment, disposable materials and the hands of medical personnel. SMA was cultured from the inner wall of the fiberoptic bronchoscope. Excessive bacterial colonies were also present in cultures of samples taken from the cleaning tank water pipe and the enzyme used for tank cleaning (Table 2). Drug susceptibility testing showed that SMA on the inner wall of the fiberoptic bronchoscope was sensitive to the compound sulfamethoxazole, minocycline, levofloxacin, mildly sensitive to chloramphenicol, ceftazidime and resistant to methicillin/clavulanic acid; similar to the drug sensitivity profile of SMA isolated from alveolar lavage fluid. Because the Microbiology Room in our hospital had been unable to perform bacterial homology analysis, we could not tell SMA contamination was from the fiberoptic bronchoscope or the alveolar lavage fluid. Thus, fiberoptic bronchoscope could only be suspected to be one of the sources of SMA contamination.

**Table 2:** Results of environmental hygiene monitoring

Monitoring items	No. of Samples	No. (Excessive colonies)	No. of SMA + Specimens
outer wall of FB	2	0	0
Inner wall of FB	3	3	3
Environmental hygiene of FB adaptor	2	0	0
Environmental hygiene of cleaning brush	2	0	0
Enzyme sterilization	3	1	0
Glutaraldehyde sterilization	4	0	0
First wash tank	2	0	0
Second wash tank	2	2	0
Last wash tank	2	2	0
Decontamination of disposable equipment	6	0	0
Monitoring finger interface	2	0	0
Bed rail	1	0	0
Bedside wall	1	0	0
Medical personnel’s hand hygiene	6	3	0

*FB:* fiberoptic bronchoscope

### Measures taken to prevent nosocomial infections

**Fig. 3:**
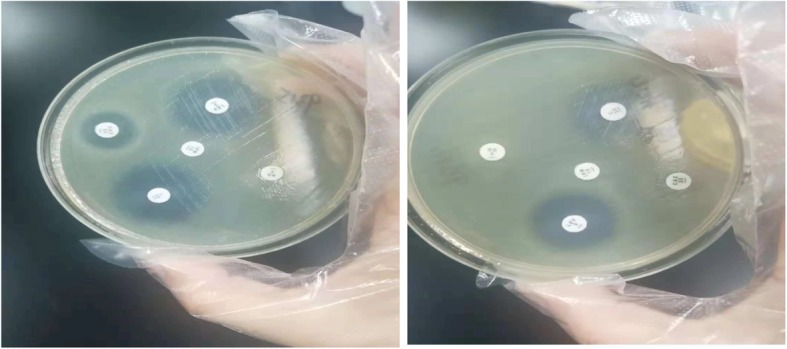
SMA drug sensitivity testing. The drug sensitivity test was performed for SMA from the patient’s alveolar lavage fluid. The zone of inhibition of rifampicin was 0, indicating that rifampicin did not inhibit the growth of Stenotrophomonas maltophilia. Two strains of Stenotrophomonas maltophilia were resistant to rifampin. RD, rifampicin; LEV, levofloxacin; C, chloramphenicol; CAZ, ceftazidime

While no patients exhibited symptoms of infection, the identification of SMA in 25 cases of alveolar lavage fluid was reported to the hospital Division of Infection. It was subsequently determined that inadequate decontamination of the fiberoptic bronchoscope was responsible. Relevant experts were immediately consulted and detailed prevention and control measures were developed and implemented. Follow-up investigations revealed that during the use of the fiberoptic bronchoscope, the 25 patients were undergoing anti-tuberculosis and anti-infection treatments with the exception of one patient who had just completed anti-tuberculosis treatment. It was reported that rifampicin combined with colistin or chloramphenicol can effectively inhibit the growth of SMA [3, 4]. We found, however, that rifampicin did not inhibit the growth of two SMA strains (Fig. 3). Based on findings from the investigation the following measures were taken: 1) use of the contaminated fiberoptic bronchoscope was terminated; 2) training in fiberoptic bronchoscope cleaning and decontamination was strengthened; 3) specific personnel were assigned to clean the fiberoptic bronchoscope, especially the inner wall component; 4) access to equipment was limited to trained personnel who were required to promptly disinfect equipment after use; 5) training in and supervision of hand hygiene were strengthened; 6) intensive decontamination of clinical work areas and clothing. Following these measures, no new incidents of SMA contamination were found over a 2-week monitoring period.

## Discussion

SMA, the sole species in the genus *Stenotrophomonas*, is a major conditional pathogen and agent of nosocomial infection. SMA infection rates are higher for elderly patients undergoing invasive procedures such as tracheotomy, endotracheal intubation and ventilator assisted ventilation. China’s CHINET annual drug resistance monitoring data for 2011 showed SMA accounting for 4.45% of all gram-negative bacteria and 11.61% of non-fermentative bacteria [5]. SMA is highly resistant to most antibacterial drugs used in clinical practice. Recently, with the widespread use of broad-spectrum antibacterial drugs and the implementation of invasive diagnostic techniques, both the incidence of SMA infections and drug resistance has increased every year [6].

The Division of Infection’s 55.55% incidence of SMA in bronchial lavage fluid was significantly higher (*p* < 0.01) than that found in other divisions during the same period and was promptly reported. A comprehensive investigation concluded that the SMA detected in samples resulted from incomplete cleaning and decontamination of the fiberoptic bronchoscope. Although patients did not exhibit symptoms of infection, the obvious safety risks triggered appropriate measures to prevent reoccurrence.

Drug sensitivity testing showed highest SMA sensitivity to minocycline, levofloxacin and chloramphenicol. This was followed by the compound sulfamethoxazole, with 92.31% sensitivity. The 2015–2016 report on drug resistance monitoring of China [7] showed SMA most sensitive to minocycline, followed by the compound sulfamethoxazole, levofloxacin, and chloramphenicol; very similar to the drug sensitivity test results described here. By contrast, the reported resistance rate of SMA to ceftazidime was 54.2%, while we found 100% resistance in our study. In the cases described here none of the patients exhibited symptoms of infection, presumably because all were undergoing anti-infective treatment with levofloxacin and other antibiotics effective in controlling SMA. It remains unclear whether anti-tuberculosis treatment also had inhibitory effects on SMA. Previous studies showed that rifampicin can effectively increase antibacterial effects on multi-drug resistant SMA, and has proven to be of important clinical significance [3, 4, 7, 8]. We note that in the event of an outbreak of clinical SMA infections, an effective treatment regimen can be promptly initiated, such as using minocycline, levofloxacin to which SMA is 100% sensitive. In the epidemiological study of nosocomial infection or bacterial contamination, it is critical to determine the routes of transmission in order to take effective measures to prevent the outbreak of clinical infections. Therefore, microbial identification and strain homology analysis are increasingly demanded, and to understand the bacterial types, subtypes and strains at the molecular levels is of great importance. Fiberoptic bronchoscope technology has evolved as a means of diagnosis and treatment. Clinical applications of fiberoptic bronchial lavage and drug delivery have expanded, especially for patients with pulmonary infections. Sputum and inflammatory secretions of target lesion can be effectively removed and small airway obstruction can be quickly alleviated. Meanwhile, deep collection of sputum samples can be quickly and accurately performed yielding strong evidence for antibiotic selection, which can effectively improve symptoms and prognosis. Frequent clinical use places stringent demands on cleaning and decontamination of fiberoptic bronchoscope equipment. Common causes of nosocomial infections associated with use of fiberoptic bronchoscopes include environmental contamination, incomplete cleaning and decontamination, improper operation, defects in endoscope dispersion cleaning and decontamination and bacterial contamination of equipment. In the cases reported here, SMA isolated from bronchial lavage fluid and the inner wall of the fiberoptic bronchoscope showed similar drug-resistant profiles, suggesting the inner wall of the bronchoscope as the likely source of bacteria, likely associated with inadequate equipment cleaning and sterilization. Specific personnel had not been assigned to clean and disinfect the fiberoptic bronchoscope, although this task was recognized as requiring a high degree of stringency. On-site sampling revealed bacteria were present in the cleaning tank, enzymes and on the hands of personnel. SMA was also directly isolated from the inner wall of the fiberoptic bronchoscope. Based on these findings, it was concluded that incomplete cleaning and decontamitation of the fiberoptic bronchoscope was responsible for the presence of SMA presenting risk of nosocomial infection. In the absence of stringent cleaning after use, a biofilm may form in the scope lumen that prevents effective removal of SMA and causes persistent infection [9]. Controlling temperature and environmental pH can prevent biofilm formation [10].

The Division of Infection conducted professional training on cleaning and decontamination of fiberoptic bronchoscopes, and strengthened training in hand hygiene, drug resistance, decontamination and isolation. Specific personnel were assigned responsibility for cleaning the bronchoscope. All bronchoscopes and other endoscopes used in other divisions were thoroughly investigated, without finding evidence of bacterial contamination.

## Conclusions

Following comprehensive measures taken, the suspected nosocomial contamination and transmission risk was effectively controlled.

## Data Availability

The datasets are available from the corresponding author under reasonable request.

## Abbreviations

BALF: Bronchoscope lavage fluid
CAZ: Ceftazidime
CL: Chloramphenicol
CLSI: Clinical Laboratory Standards
IPM: Imipenem
LVX: Levofloxacin
MH: Minocycline
SMA: *Stenotrophomonas maltophilia*
SXT: Sulfamethoxazole
TIM: Ticarcillin/clavulanic acid
